# *Aiouea padiformis* extract exhibits anti-inflammatory effects by inhibiting the ATPase activity of NLRP3

**DOI:** 10.1038/s41598-024-55651-z

**Published:** 2024-03-04

**Authors:** Sumin Lee, Qianying Ye, Hyeyun Yang, Sojung Lee, YeJi Kim, Nahyun Lee, Darwin Gonzalez-Cox, Dong-Keun Yi, Soo-Yong Kim, Sangho Choi, Taesoo Choi, Man S. Kim, Seong Su Hong, Chun Whan Choi, Yoonsung Lee, Yong Hwan Park

**Affiliations:** 1 Department of Microbiology, Ajou University School of Medicine, Suwon, 16499 Republic of Korea; 2 Department of Biomedical Science and Technology, Kyung Hee University, Seoul, 02447 Republic of Korea; 3 Department of Biomedical Sciences, Graduate School of Ajou University, Suwon, Republic of Korea; 4 Clinical Research Institute, Kyung Hee University Hospital at Gangdong, School of Medicine, Kyung Hee University, Seoul, 05278 Republic of Korea; 5 Herbarium of National Autonomous University of Nicaragua at Leon, Leon, 21000 Nicaragua; 6 International Biological Material Research Center, Korea Research Institute of Bioscience and Biotechnology, Daejeon, 34141 Republic of Korea; 7 Department of Urology, School of Medicine, Kyung Hee University, Seoul, 05278 Republic of Korea; 8 Natural Product Research Team, Gyeonggi Bio-Center, Suwon, Republic of Korea

**Keywords:** NLRP3 inflammasome, Anti-inflammation, Plant extracts, *Aiouea padiformis*, Lauraceae, Biochemistry, Cell biology, Drug discovery, Immunology, Molecular biology

## Abstract

Inflammation is implicated as a cause in many diseases. Most of the anti-inflammatory agents in use are synthetic and there is an unmet need for natural substance-derived anti-inflammatory agents with minimal side effects. *Aiouea padiformis* belongs to the Lauraceae family and is primarily found in tropical regions. While some members of the *Aiouea* genus are known to possess anti-inflammatory properties, the anti-inflammatory properties of *Aiouea padiformis* extract (AP) have not been investigated. In this study, we aimed to examine the anti-inflammatory function of AP through the NOD-, LRR- and pyrin domain-containing protein 3 (NLRP3) inflammasome and elucidate the underlying mechanisms. Treatment with AP inhibited the secretion of interleukin-1 beta (IL-1β) mediated by NLRP3 inflammasome in J774A.1 and THP-1 cells without affecting the viability. In addition, AP treatment did not influence NF-κB signaling, potassium efflux, or intracellular reactive oxygen species (ROS) production—all of which are associated with NLRP3 inflammasome activation. However, intriguingly, AP treatment significantly reduced the ATPase activity of NLRP3, leading to the inhibition of ASC oligomerization and speck formation. Consistent with cellular experiments, the anti-inflammatory property of AP in vivo was also evaluated using an LPS-induced inflammation model in zebrafish, demonstrating that AP hinders NLRP3 inflammasome activation.

## Introduction

Inflammation is a critical component of the innate immune system, mostly triggered by external stressors. It promotes the recovery of tissue damage induced by injury and infection; however, uncontrolled inflammation can become pathological. Since inflammation is induced by pro-inflammatory cytokines including interleukin-1β (IL-1β) and interleukin-18 (IL-18), cytokine production must therefore be carefully regulated to reduce inflammation^1^. IL-1β and IL-18 overproduction can also contribute to the development of various inflammatory diseases^2^. Therefore, understanding the mechanisms regulating IL-1β and IL-18 secretion is crucial for addressing and treating these diseases, and the protein complex responsible for these secretion is called inflammasomes^3^.

The NOD-like receptor pyrin domain-containing protein 3 (NLRP3) inflammasome is the most widely studied inflammasome. Unlike other inflammasomes that need a direct recognition ligand to be activated, NLRP3 is activated by changes in intracellular homeostasis^4^. This ability allows it to respond to various alterations in the intracellular environment, contributing to the exacerbation of symptoms in many diseases. Mutations in NLRP3 lead to cryopyrin-associated periodic syndrome (CAPS)^5–7^. Moreover, hyperactivation of the NLRP3 inflammasome is associated with a variety of inflammatory diseases, such as arthritis^8,9^, diabetes^10^, Crohn's disease^11^, asthma^12^, and even neurodegenerative disorders like Alzheimer's^13^ and Parkinson's diseases^14,15^. Given the crucial role of dysregulated NLRP3 activity in these conditions, understanding the regulatory mechanisms of NLRP3 activation and identifying how to suppress the NLRP3 inflammasome are of clinical importance.

NLRP3 inflammasome activation occurs in two phases^16^. The initial phase, known as priming, mainly involves nuclear factor-kappa B (NF-κB) signaling. Pathogen-associated molecular patterns (PAMPs) like lipopolysaccharide (LPS) activate toll-like receptors, leading to the activation of NF-κB. This triggers the translocation of NF-κB into the nucleus, inducing the expression of inflammatory genes such as NLRP3 and pro-IL-1β^17,18^. The second activation phase is triggered by damage-associated molecular patterns (DAMPs) like lysosomal rupture^19^, intracellular reactive oxygen species (ROS)^20^, endoplasmic reticulum stress^21^, and potassium (K^+^) efflux^22,23^. In this phase, NLRP3 forms an inflammasome with pro-caspase-1 and apoptosis-associated speck-like protein containing a caspase recruitment domain (ASC)^24^. This leads to the cleavage of pro-caspase-1 into active caspase-1, which in turn cleaves pro-IL-1β and pro-IL-18 to active form.

Given that the factors influencing the activation of NLRP3 play crucial roles in numerous intracellular signaling pathways, inhibiting them to suppress NLRP3 could result in significant side effects. Currently, there are FDA-approved drugs that can directly inhibit IL-1β^25^. Nevertheless, inhibiting IL-1β directly increases the susceptibility to different infections, prompting continuous investigation into small molecules that specifically target NLRP3. Despite showing promising effectiveness, currently there are no FDA-approved NLRP3 inhibitors, primarily due to concerns about toxicity^26^. Natural products, due to the lower likelihood of side effects, are often preferred over synthetic chemicals, especially for the treatment and prevention of chronic diseases, where patients may require long-term medication^27^. Based on this, our research focused on identifying natural products that could effectively target the NLRP3 inflammasome, a key player involved in many chronic diseases. In the initial experiment, we performed a thorough screening of extracts from 200 different plant species found in Costa Rica and Nicaragua. Our criteria for selection included the absence of cytotoxicity, no impact on NF-kB signaling, and documented medicinal use of any plant from the same family. From this screening, we identified *Aiouea padiformis* extract (AP), which showed a specific function to suppresses NLRP3 without adverse effects.

*Aiouea padiformis* (Standl. & Steyerm.) R. Rohde, formerly known as *Cinnamomum padiforme* (Standl. & Steyerm.) Kosterm or *Phoebe padiformis* Standl. & Steyerm., belongs to the Lauraceae family^28^. It is primarily found in tropical regions of North and South America. The plants of the *Aiouea* genus have traditionally been used in various folk remedies. *Aiouea padiformis* is used as a medicinal plant to treat various ailments, such as fever, colds, and headaches^28^. *Aiouea dubia* was used to treat epilepsy^29^. Furthermore, several species within the *Aiouea* genus have been investigated for the presence of bioactive compounds with medicinal properties in plant extract studies. The essential oils from *A. maguireana* were found to contain oxygenated sesquiterpenes, which are known to have anti-inflammatory properties^30^. An ethanol extract of xylopodium from *A. trinervis* was found to contain three types of butanolides that exhibit antitrypanosomal activity^31,32^. *Aiouea montana* (Sw.) R.Rohde has an antioxidant activity and free radical-scavenging capacity, which can reduce NLRP3 inflammasome activation^33^. Furthermore, some plants from the family Lauraceae, including *Aiouea saligna* Meisn., have been shown to inhibit the release of prostaglandin E2, one of the main mediators of inflammation^34^. Although the medicinal activity and metabolomic profile of other members of the *Aiouea* genus have been investigated, the mechanisms underlying these anti-inflammatory effects remain unknown. Our research focuses on elucidating the molecular mechanisms underlying the anti-inflammatory effects exhibited by *Aiouea padiformis*. At the cellular level, we confirmed the anti-inflammatory effects of AP and observed its effects on NLRP3 inflammasome. To complement these findings, we utilized the zebrafish model to assess the anti-inflammatory effects of AP at an organismal level. Zebrafish share a similar genomic profile with humans^35^, especially in aspects related to the innate immune system, including inflammatory responses^36–38^. This makes them an ideal model for studying inflammation and testing the anti-inflammatory effects of various compounds. Consequently, our research confirms the anti-inflammatory effectiveness of AP in both cellular and whole organism context. We believe that our finding can offer novel therapeutic strategies to alleviate various NLRP3-associated diseases, while minimizing the risk of adverse effects.

## Results

### AP specifically reduces NLRP3 inflammasome activation

We screened extracts from 45 different plant species native to Nicaragua for NLRP3 inhibition activity. The reduction in IL-1β secretion was assessed by immunoblotting and the relative expression was quantified using ImageJ (ver. 1.54d) (Supplemental Table 1). Among the 45 plant extracts tested, we selected candidates that did not affect cell viability, showed concentration-dependent reduction in IL-1β secretion, and did not inhibit the NF-κB signal. Among these selected candidates, AP appeared to effectively reduce IL-1β and was therefore used for subsequent experiments.

**Figure 1:**
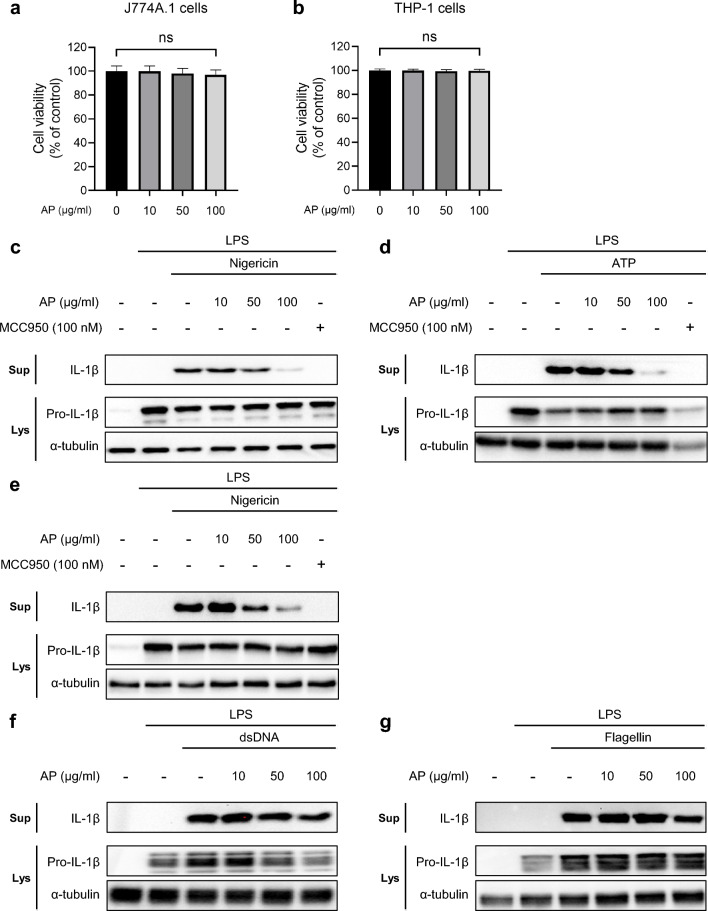
AP specifically reduces NLRP3 inflammasome activation. (**a,b**) Cell viability assays of J774A.1 cells (**a**) and differentiated THP-1 cells (**b**) treated with AP (10 μg/mL, 50 μg/mL, 100 μg/mL) for 2 h. Cell viability was analyzed by EZ-CYTOX, measured at a wavelength of 450 nm. *Ns* not significant. (**c–e**) Western blots of IL-1β in the supernatants (Sup), pro-IL-1β and α-tubulin in the soluble lysates (Lys) of treated cells. LPS-primed J774A.1 cells were treated with AP for 2 h and activated for 30 min with nigericin (10 μM) (**c**) or ATP (5 mM) (**d**) with or without MCC950, differentiated, and LPS-primed THP-1 cells were treated with AP for 2 h and activated for 30 min with nigericin (10 μM) with or without MCC950 (**e**). (**f,g**) LPS-primed J774A.1 cells were treated with AP for 2 h and activated for 3 h with transfection of dsDNA (2 μg/mL) (**f**) or flagellin (1.25 μg/mL) (**g**).

To confirm the anti-inflammatory effects of AP, we first measured the viability of mouse J774A.1 and human THP-1 cells after AP treatment. The viability of both types of cells was not altered even when treated with the highest concentration of 100 μg/mL of AP (Fig. 1a, b). We then investigated the effect of AP treatment on IL-1β secretion mediated by NLRP3 inflammasome. When LPS-primed J774A.1 cells were exposed to nigericin or adenosine triphosphate (ATP), well-known NLRP3 activators, they showed a significant increase in IL-1β secretion. However, when the cells were treated with both the activators and AP, IL-1β and IL-18 secretion decreased in a concentration-dependent manner (Fig. 1c, d, Supplemental Fig. 1). The effects of AP were compared with those of MCC950, an NLRP3-specific inhibitor^39^. As shown in Fig. 1c, d, a high concentration of AP inhibited NLRP3 activation to a similar extent as MCC950. Similar results were obtained when phorbol 12-myristate 13-acetate (PMA)-primed THP-1 cells were treated with nigericin alone or in combination with AP or MCC950 (Fig. 1e). Lastly, to confirm whether AP selectively inhibits NLRP3 or also affects other inflammasomes, we activated Absent in Melanoma 2 (AIM2) and NOD-like receptor family CARD domain-containing protein 4 (NLRC4) inflammasomes by treating the cells with dsDNA or flagellin, respectively, followed by AP treatment. When we measured the secretion of IL-1β, AP treatment did not affect IL-1β production, indicating that AP specifically blocked the activation of the NLRP3 inflammasome (Fig. 1f, g).

### AP does not impact the NF-κB signaling pathway

**Figure 2:**
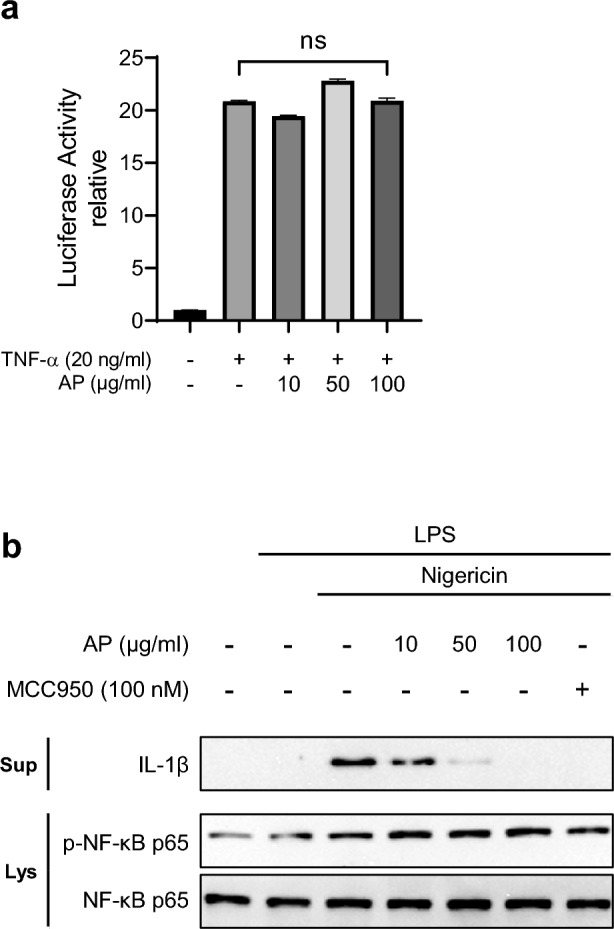
AP does not affect the NF-κB signaling pathway. (**a**) 293 T cells were transfected with the pGL4.32 luciferase reporter vector and treated with TNF-α (20 ng/mL) for 5 h with or without AP. Luciferase activity was analyzed using the Promega Bright-Glo™ Luciferase Assay System. (**b**) LPS-primed J774A.1 cells were treated with AP for 2 h and activated for 30 min with nigericin (10 μM). IL-1β in the supernatants (Sup), phospho-NF-κB and NF-κB in the soluble lysates (Lys) were analyzed using a Western blot.

We then investigated how AP suppresses the NLRP3 inflammasome. The production of IL-1β involves two steps: the priming phase, activating NF-κB to increase pro-IL-1β transcription, and the activation phase, inducing inflammasome formation. To check if AP reduces NF-κB activity during the priming step, we assessed NF-κB activity by an NF-κB luciferase reporter gene assay. Treatment with tumor necrosis factor-alpha (TNF-α) significantly increased NF-κB signaling, and co-treatment with TNF-α and AP had no effect on the elevated NF-κB signaling (Fig. 2a). To further confirm these findings, we analyzed phospho p65 levels, an NF-κB activity indicator. AP treatment did not affect the levels of phosphorylated p65 (Fig. 2b). These results indicate that AP suppresses the NLRP3 inflammasome without affecting NF-κB signaling.

### AP suppression of the NLRP3 inflammasome is not dependent on K^+^ efflux, increased intracellular ROS level, or mitochondrial membrane potential

**Figure 3:**
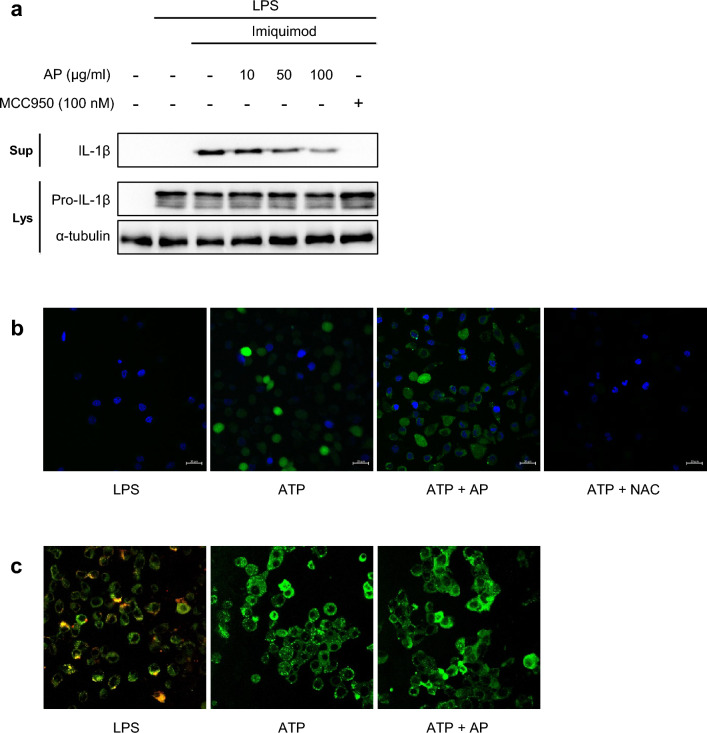
AP inhibits NLRP3 inflammasome, regardless of K+ efflux, intracellular ROS, and mitochondrial membrane potential. (**a**) LPS-primed J774A.1 cells were treated with AP for 2 h and activated with Imiquimod (200 μM) for 1 h. (**b,c**) LPS-primed J774A.1 cells treated with AP (50 μg/mL) for 2 h and activated with ATP (5 mM) for 5 min. Representative immunofluorescence images of ROS (DCFDA; green color) (**b**) and mitochondrial membrane potential (JC-1 monomer; green color, J-aggregates; red color) (**c**) were taken by a confocal laser-scanning microscope (Carl Zeiss, LSM710, scale bar, 20 μm).

As AP did not have any influence on the priming step, we investigated its potential impact on the activation step. In the process of activating the NLRP3 inflammasome, two key events are the efflux of potassium ions or an increase in intracellular ROS. Blocking these events can suppress NLRP3 inflammasome activity. To explore if AP could hinder NLRP3 activation by affecting potassium efflux, we treated cells with imiquimod, which activates the NLRP3 inflammasome independently of K^+^ efflux^40^. As shown in Fig. 3a, AP suppressed NLRP3 activation by imiquimod in a concentration-dependent manner, suggesting that the inhibition of the NLRP3 inflammasome by AP is not dependent on K^+^ efflux. To determine whether AP regulates intracellular ROS levels to inhibit the NLRP3 inflammasome, we measured ROS levels after AP treatment. While ATP treatment increased ROS levels, AP did not influence intracellular ROS level. However, when cells were treated with N-acetylcysteine (NAC), a well-known ROS scavenger, ROS levels significantly decreased (Fig. 3b). NLRP3 activators can induce mitochondrial instability, and the resulting mitochondrial dysfunction may lead to the activation of the NLRP3 inflammasome. Therefore, we examined alterations in mitochondrial function by measuring the mitochondrial membrane potential in cells treated with ATP, followed by AP or not. The decrease in the ratio of red to green fluorescence intensity indicates mitochondrial depolarization. While mitochondrial membrane potential exhibited a significant alteration with ATP treatment, AP treatment did not mitigate the change in membrane potential (Fig. 3c). Therefore, we found that K^+^ efflux, intracellular ROS, and mitochondria are not implicated in the mechanism by which AP inhibits the activation of the NLRP3 inflammasome.

### AP inhibits ATPase activity and ASC oligomerization of the NLRP3 inflammasome

**Figure 4:**
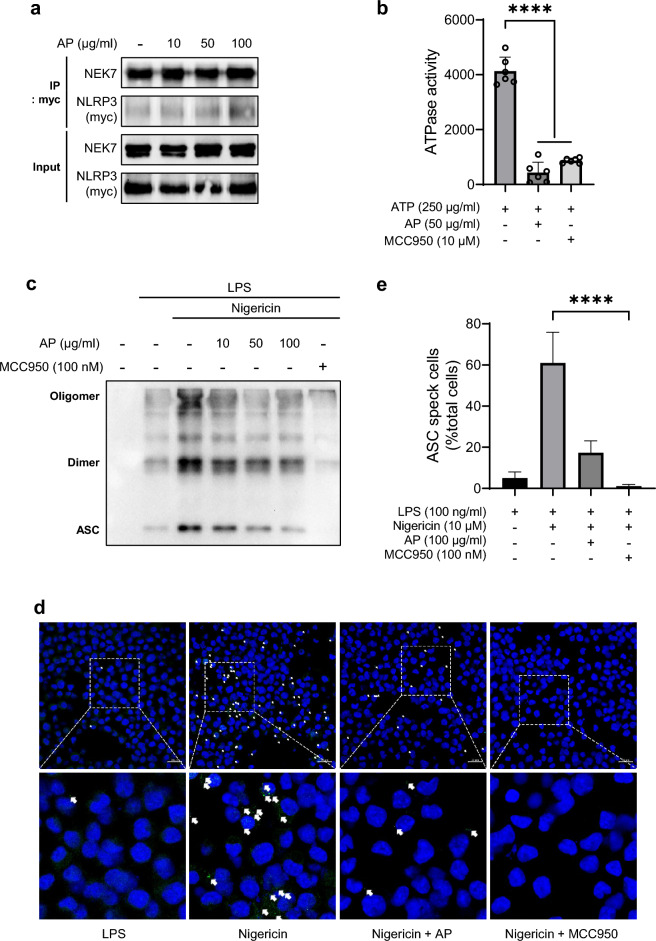
AP blocks NLRP3 inflammasome assembly by hindering ATPase activity of NLRP3. (**a**) HEK 293FT cells were transfected with NLRP3-Myc and treated with or without AP for 2 h. The interaction between NLRP3 and NEK7 was analyzed by immunoprecipitation and immunoblot. (**b**) The ATPase activity of NLRP3 with or without AP was measured by luminescence using the ADP-Glo™ Max assay. (**c**) LPS-primed THP-1 cells were treated with AP for 2 h and activated with nigericin (10 μM) for 30 min. The cell pellet was cross-linked by DSS (2.5 mM) for 30 min. ASC oligomerization was analyzed by immunoblot. (**d,e**) LPS-primed J774A.1 cells treated with AP for 2 h and activated with nigericin (10 μM) for 30 min. Representative immunofluorescence images of ASC speck formation (indicated by arrows) were taken by a confocal laser-scanning microscope (Carl Zeiss, LSM710, scale bar, 20 μm) (**d**). Graph representing the number of cells containing ASC specks in all treatments (**e**).

Never in Mitosis Gene A-related kinase 7 (NEK7) is essential for promoting oligomerization of the NLRP3 inflammasome, which is necessary for its activation^41,42^. We examined the effect of AP on the interaction between NEK7 and the NLRP3 using co-immunoprecipitation experiment. No significant differences in the amount of NEK7-NLRP3 complexes were observed between the untreated samples and those treated with AP. Therefore, we confirmed that AP did not interfere with the interaction of NEK7 and NLRP3 (Fig. 4a).

ATPase activity is essential for the assembly of the NLRP3 inflammasome^43^. Many NLRP3 inhibitors are known to impede the ATPase activity of NLRP3^44,45^; therefore, we set out to determine whether AP affects ATPase activity. As shown in Fig. 4b, AP significantly reduced the ATPase activity of NLRP3, and the extent of reduction was nearly identical to the well-known ATPase activity inhibition by MCC950. We subsequently investigated ASC oligomerization, a marker of inflammasome assembly^46^. Since the ATPase activity of NLRP3 is crucial for inflammasome oligomerization, it can be predicted that the oligomerization of ASC, a common component of the inflammasome, will also be inhibited by AP. As expected, AP treatment attenuated ASC oligomerization in a concentration-dependent manner (Fig. 4c). These observations were further confirmed by assessing the extent of ASC speck formation using confocal microscopy, which represents inflammasome assembly. Consistent with previous data, speck formation was also significantly reduced by AP (Fig. 4d, e). These results revealed that the AP-mediated inhibition of NLRP3’s ATPase activity can be attributed to the prevention of inflammasome assembly.

To identify the major bioactive component in AP responsible for the IL-1β inhibitory activity, AP was subjected to MPLC. Supplemental Fig. 2a shows the identification of the main chromatography of AP. Among the AP fractions (AP1-AP11), the AP7 fraction was found to contain the most abundant components (Supplemental Fig. 2b). Consequently, the AP7 fraction was subjected to recycle-HPLC, resulting in the isolation of five fractions (AP71-AP75). Among them, Compound 1 (3.1 mg, tR = 54.1 min) was isolated from subfraction AP75 using preparative HPLC (acetonitrile–water, 15:85 to 85:15 in 90 min). The structure of the isolated compound was elucidated by MS and 1D/2D NMR data analyses and was confirmed to be diethyl phthalate upon compared with the corresponding data reported in the literature (Supplemental Fig. 2a).

### AP alleviates inflammatory responses induced in the zebrafish embryos

To evaluate the anti-inflammatory effects of AP at the organism level, we used a zebrafish (*Danio rerio*) model. Zebrafish embryos are an effective tool to study inflammation during early development^47,48^. Inflammation can be induced through various methods, including LPS immersion, direct injection of LPS into the yolk sac, and tail-cut injuries^36^.

**Figure 5:**
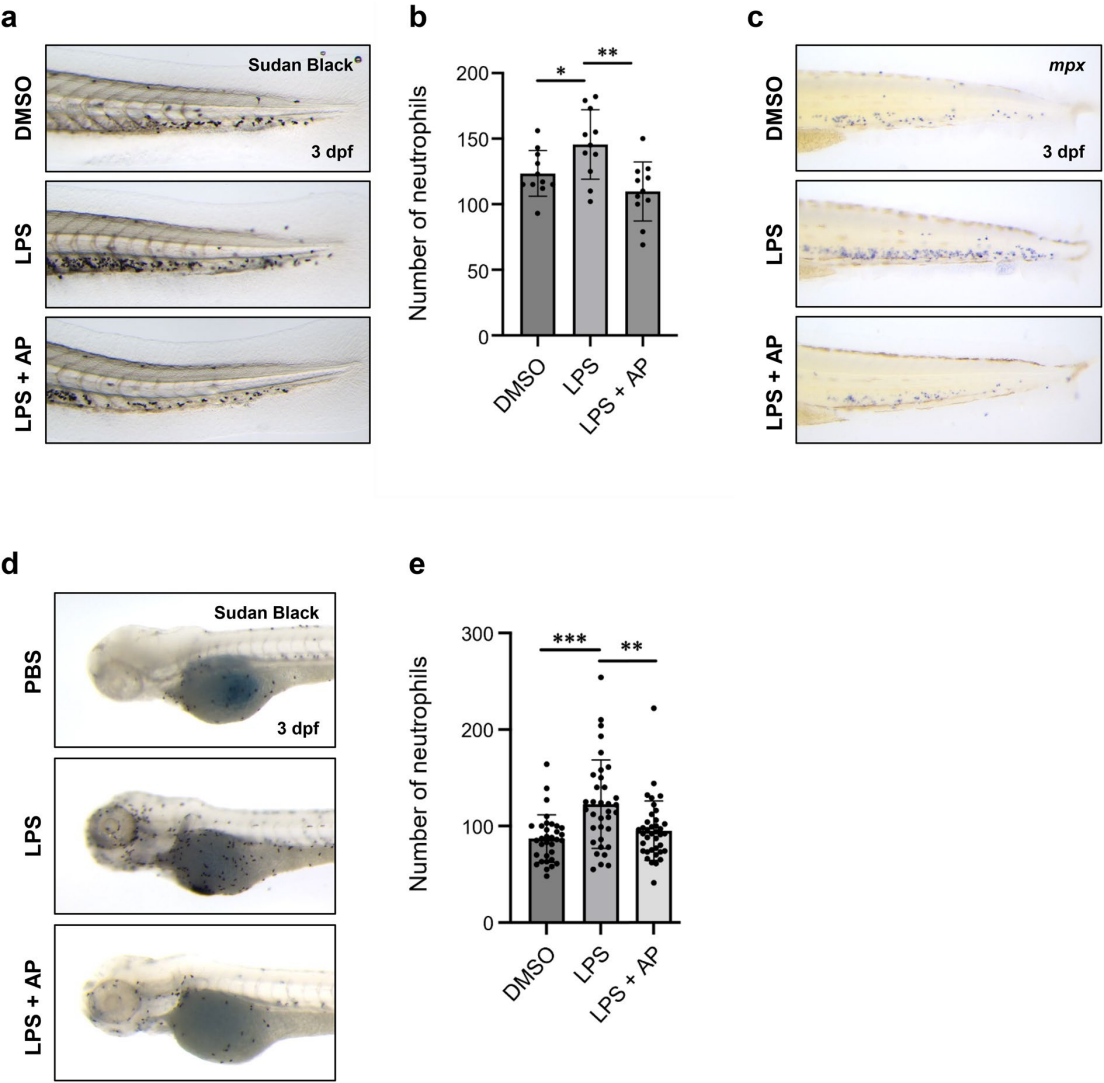
AP alleviates inflammatory response induced by LPS treatment in zebrafish. (**a**) Representative images of posterior areas of Sudan Black B-stained zebrafish embryos after LPS induction and AP treatment at 3 dpf. The upper panel shows the control group with DMSO (0.025%) treatment, the middle panel shows LPS inflammation group, and the bottom panel shows the LPS inflammation group after treatment with 10 µg/mL AP. (**b**) Quantification of neutrophils in the posterior region of zebrafish after LPS induction and AP treatment. (**c**) Representative images of the caudal hematopoietic tissues after LPS induction and AP treatment at 3 dpf. Whole-mount in situ hybridization was conducted using an *mpx* probe. (**d**) Representative images of anterior Sudan Black B-stained zebrafish embryos following direct LPS injection and AP treatment at 3 dpf. (e) Quantification of the number of neutrophils in the anterior zebrafish embryos after LPS induction and AP treatment. All graphs represent the mean ± S.E.M. of individual values. P-values were calculated using an unpaired two-tailed Student’s t-test. *P < 0.05; **P < 0.01; ***P < 0.001.

We conducted a primary experiment by inducing inflammation in 1 day post-fertilization (dpf) zebrafish embryos by treating the larvae with 10 µg/mL LPS. Following an induction period of 2 days, the embryos were fixed. The resulting inflammatory response was assessed by monitoring the number of myeloid cells using Sudan Black B staining^49^. Inflammation was successfully induced in the LPS-treated zebrafish, and co-treatment with 10 µg/mL of AP markedly reduced this inflammation (Fig. 5a). Further quantification revealed a significant reduction in the levels of the inflammatory markers in embryos treated with AP compared to those that were not (Fig. 5b). Additionally, under the same conditions, whole-mount hybridization (WISH) analysis targeting neutrophil-specific *mpx* expression revealed that AP treatment reduced the LPS-induced increase in *mpx*-positive cells (Fig. 5c). In addition to the LPS-immersion experiments, we induced inflammation by directly injecting LPS into the zebrafish yolk. This approach led to noticeable inflammation, particularly around the injection site and the anterior part of the embryos. The enhanced inflammatory response was significantly alleviated by incubation with AP (Fig. 5d, e).

Taken together, the findings of our experiments on the zebrafish model verify the potent anti-inflammatory effects of AP at the organismal level, further emphasizing the broader potential of this natural product as an anti-inflammatory agent.

## Discussion

Elevated IL-1β expression is involved in many diseases, and the severity of these diseases is often correlated with the magnitude of IL-1β upregulation^1^. Consequently, strategies aimed at blocking IL-1β have emerged as promising therapeutic approaches for inflammatory diseases. IL-1β secretion is intricately controlled by various inflammasomes, requiring research to understand the regulation of these inflammasomes and for the development of inhibitors. While most inflammasomes directly recognize DAMPs or PAMPs, NLRP3 detects cellular homeostatic changes, without direct recognition of specific components, thereby contributing to the pathogenesis of various diseases where such alterations are observed, and also creating a feedback loop that exacerbates disease symptoms. Given the association of NLRP3 with numerous diseases, ongoing research is actively exploring technologies specifically designed to inhibit NLRP3. While ongoing research actively explores small molecules directly inhibiting NLRP3, none of these compounds has yet received FDA approval due to concerns about their toxicity. To address these safety concerns, extensive efforts are being made to investigate natural products as potential alternative NLRP3 inhibitors.

Therefore, we examined the anti-inflammatory properties of about 200 different plant extracts from Costa Rica and Nicaragua which had not been studied before. Among the investigated extracts, we identified potent NLRP3 inhibitory effects in *Guarea*^50^ and *Trichospira* from Costa Rica, and these findings have been recently submitted for publication. Given that among the 45 Nicaraguan compounds tested, AP exhibited the highest level of anti-inflammatory activity, and considering the numerous studies reporting anti-inflammatory effects in plants belonging to the same genus, we believe it is worthwhile to investigate the anti-inflammatory function of AP. In this study, we aimed to demonstrate the inhibitory effect of AP on NLRP3 inflammasome activation and elucidate its underlying mechanism. As recently developed NLRP3 inhibitors, such as MCC950, exert their mechanism through the inhibition of NLRP3's ATPase activity^39^, thereby disrupting the assembly of the NLRP3 inflammasome and many NLRP3 inhibitors currently under development also target the ATPase activity of NLRP3, we also investigated whether AP could similarly inhibit the ATPase activity of NLRP3, leading to the decreased ASC oligomerization and speck formation. As expected, AP effectively suppressed the ATPase activity (Fig. 4c–e). Fundamentally, to inhibit ATPase activity, it is necessary to directly bind to NLRP3. Therefore, we analyzed the composition of the extracts using HPLC to identify which components of AP bind to NLRP3. The HPLC analysis of AP revealed that diethyl phthalate is the most abundant component in AP. Diethyl phthalate is commonly used in the manufacturing of plastics, insecticides, cosmetics, and aspirin. In addition, since it often used as plasticizers for polyvinyl, polyvinyl chloride, or cellulose resins, there have been reports on the potential risks associated with long-term exposure to it^51–55^. While there are studies suggesting that diethyl phthalate extracted from plants might be artificial due to contamination in various containers or during the extraction process^56,57^, many studies have indicated its extraction from bacteria and plants, highlighting its anti-inflammatory properties^58,59^. However, in this study, as we could not confirm whether diethyl phthalate can modulate the ATPase activity of NLRP3, it is essential to investigate whether various substances collaborate to inhibit NLRP3 or if they can individually inhibit NLRP3. Additionally, the impact of each isolated compound on cells should be determined. In addition, it is important to identify the substances among the discovered active compounds that directly bind to NLRP3, inhibiting its ATPase activity. Future research involving extensive collection of AP could allow for the analysis of additional substances within AP. This could lead to the discovery of new compounds directly binding to NLRP3 or investigating various activities of diethyl phthalate.

Our study suggests the potential of AP as an NLRP3 inhibitor; however, many questions remain. First, as the composition of AP may be influenced by environmental factors, the separation and purification of bio active substances are challenging due to the time required for large-scale collection under the same conditions. However, securing the confirmed active compounds is expected to be a crucial starting point in the search for NLRP3 inhibitors from plant extracts. Second, in our experiment, AP significantly reduced the ATPase activity of NLRP3 and ASC speck formation but did not regulate its binding with NEK7 (Fig. 4). However, the association of NEK7 with NLRP3 is the essential for the assembly of the NLRP3 inflammasome, including ASC speck formation^24^. Recently, several studies have suggested that the phosphorylation of NLRP3 is crucial for the activation of the NLRP3 inflammasome^60–62^. In addition, recent studies have reported that NLRP3 exists in an inactive CAGE form^63^. Therefore, additional research is essential to verify whether AP influences phosphorylation or preserves the CAGE model, thereby impeding activation. Third, as gain-of-function mutations in NLRP3 gene like R260W, D303G, and E311K cause autoinflammatory disease, CAPS, we need to assess the potential utilization of newly discovered substances as a treatment for CAPS patients by determining where they bind on NLRP3. In the case of the well-known NLRP3 inhibitor MCC950, it had no effect on CAPS patients with specific mutations^64^. Therefore, it is crucial to identify the binding location of the substances we have discovered on NLRP3 and understand how they function in CAPS patients. Finally, the medicinal use of *Aiouea* species is well-known, and some species have been analyzed for their constituent components. However, there is currently a lack of substantial scientific evidence regarding their potential medicinal properties, especially in terms of demonstrating strong anti-inflammatory activity. To determine whether specific medicinal uses are associated with *Aiouea* species, it is necessary to explore detailed research or traditional practices specific to each species.

## Conclusion

These results show that AP suppresses ATP hydrolysis through the NACHT domain of NLRP3, which possesses ATPase activity, thereby blocking conformational changes in NLRP3. Our results suggest that compounds in the AP may bind directly to the NACHT domain of NLRP3, thus inhibiting its ATPase activity. Overall, this study could provide insight into the anti-inflammatory properties of AP and potentially lead to the development of novel anti-inflammatory therapies for disorders involving NLRP3 inflammasome activation.

## Materials and methods

### Reagents and antibodies

*Aiouea padiformis* (Standl. & Steyerm.) R. Rohde was provided by Korea Research Institute of Bioscience and Biotechnology; KRIBB. Dulbecco’s modified Eagle medium (DMEM), Roswell Park Memorial Institute (RPMI) 1640, penicillin–streptomycin, fetal bovine serum (FBS), pure down protein A/G-agarose (P9203-200), and phosphatase inhibitor cocktail (P3200-005) were purchased from GenDEPOT (Baker, TX, USA). The protease inhibitor cocktail (PPI1015) was purchased from Quartett GmbH (Berlin, Germany). Antibodies against α-tubulin (sc-5286), NIMA-related kinase 7 (NEK7; B-5) (sc-393539), c-Myc (9E10) (sc-40) and ASC (sc-514414) were purchased from Santa Cruz Biotechnology (Santa Cruz, CA, USA). Human IL-1β (AF-401-NA), mouse IL-1β (AF-201-NA) antibodies, and mouse IL-18 DuoSet ELISA kit (DY7625-05) were purchased from R&D Systems (Minneapolis, MN, USA). Anti-Caspase-1 (A0964) was purchased from Abclonal Technology (Woburn, MA, USA). Phospho-NF-κB p65 (93H1), NF-κB p65 (8242) antibodies were purchased from Cell Signaling Technology (Danvers, MA, USA). Lipopolysaccharide from *P. gingivalis* (LPS, Ultrapure) (tlrl-ppglps), nigericin (tlrl-nig), ATP (tlrl-atpl), and flagellin (FLA-ST; tlrl-epstfla) were purchased from InvivoGen (San Diego, CA, USA). Highly cross-adsorbed Mouse IgG (H + L) secondary antibodies (A32723), MitoProbe™ JC-1 Assay Kit (M34152), Lipofectamine™ 2000 (11668019) and 3000 (L3000015) were purchased from Invitrogen (Thermo Fisher Scientific, Waltham, MA, USA). Phorbol 12-myristate 13-acetate (PMA; HY-18739) and Imiquimod (IMQ; HY-B0180) were purchased from MedChem Express (South Brunswick Township, NJ, USA). EZ-CYTOX (EZ-500) was purchased from DoGenBio (Shanghai, China). *N*-acetyl-l-cysteine (NAC; A9165-25G) and disuccinimidyl suberate (DSS; 68528-80-3) were purchased from Sigma-Aldrich (St Louis., MO, USA). The cellular reactive oxygen species (ROS) assay kit (DCFDA/H2DCFDA) (ab113851) and nuclei stained with mounting medium (DAPI) (ab104139) were purchased from Abcam (Cambridge, UK). Bright-Glo™ Luciferase assay system (E2610), pGL4.32 Luciferase Reporter vector (E8491), and ADP-Glo™ Max Assay (V7001) were purchased from Promega (Madison, WI, USA).

### Plant sample collection

The collection and preservation of plants material were conducted under the agreement for sustainable exploitation of the Nicaraguan flora between International Biological Material Research Center (IBMRC) in Korea Research Institute of Bioscience and Biotechnology (KRIBB) of Korea and Universidad Nacional Autonoma de Nicaragua-Leon of Nicaragua. All research samples follow the regulation of Nicaragua institutional, national, and international guidelines and legislation, with all necessary permissions and licenses obtained to ensure compliance with ethical and legal requirements.

### Zebrafish

All experimental protocols and procedures involving the embryos were approved by the Institutional Animal Care and Use Committees at Kyung Hee University Hospital at Gangdong (IACUC: KHNMC AP 2023) and were performed in accordance with approved guidelines.

### In vitro study

#### Cell culture and stimulation

THP-1 and J774A.1 cells (Korean Cell Line Bank, passage numbers between 5 and 15) were collected and cultured as previously described (ATCC). We treated PMA (500 nM) for 3 h to differentiate THP-1 cells, and incubated cells for 2 days. J774A.1 cells were incubated for 1 day. Subsequently, we treated LPS (100 ng/mL) for 5 h in the cells for priming. After the cells were primed with LPS for 3 h, AP (10 μg/mL, 50 μg/mL, 100 μg/mL) was further treated for 2 h. To activate NLRP3 inflammasome, we treated Nigericin (10 μM) or ATP (5 mM) for 30 min or 1 h, or Imiquimod (200 μM) for 1 h in the cells. To activate AIM2 or NLRC4 inflammasome, we transfected dsDNA (2 μg/mL) or flagellin (1.25 μg/mL) for 3 h using Lipofectamine™ 2000. IL-1β in the supernatants, and pro-IL-1β and α-tubulin in the soluble lysates were analyzed by immunoblotting.

#### In vitro cell viability assay

In a 96-well plate, differentiated THP-1 cells (2 × 10^5^ cells/well) were seeded 2 days before the experiment. J774A.1 cells (1 × 10^5^ cells/well) were seeded 1 day before the experiment. The cells were treated with AP (10 μg/mL, 50 μg/mL, 100 μg/mL) for 2 h. After treatment, we treated the cells with EZ-CYTOX for 30 min (J774A.1) or 1 h (THP-1). The absorbance was measured at 450 nm wavelengths using an iMark™ Microplate Absorbance Reader (1681130; Bio-Rad Laboratories, Hercules, CA, USA).

#### NF-κB luciferase activity reporter gene assay

In a 96-well white plate, 293 T cells (0.2 × 10^5^ cells/well) were seeded 1 day before the transfection. The cells were transfected with pGL4.32 Luciferase Reporter Vector (0.1 μg/well) using Lipofectamine™ 3000. After 24 h, NF-κB signaling was activated by TNF-α (20 ng/mL) for 5 h with or without AP (10 μg/mL, 50 μg/mL, 100 μg/mL). Luciferase activity assay was conducted using a Bright-Glo™ Luciferase Assay System (Promega, Madison, USA) according to the manufacturer’s protocol, using a BMG LabTech FLUOstar OPTIMA microplate reader.

#### Measurement of cellular ROS assay

In a 4-well culture slide, J774A.1 cells (2 × 10^5^ cells/well) were seeded 1 day before the experiment. Cells were primed by treating them with LPS (100 ng/mL) for 5 h, followed by AP (50 μg/mL) treatment for 2 h. The medium was removed, and the cells were washed with 1× buffer (250 μL/well). Subsequently, we treated the cells with DCFDA solution (20 μM) for 1 h at 37 ℃ in the dark. The solution was removed, and cells were washed with 1× buffer (250 μL/well), and activated with ATP (5 mM) for 5 min. The cells were mounted in a medium containing DAPI for nuclear staining. Images were obtained using a confocal laser-scanning microscope (Carl Zeiss, LSM710).

#### Measurement of mitochondrial membrane potential

J774A.1 cells (2 × 10^5^ cells/well) seeded in a 4-well culture slide primed with LPS (100 ng/mL) for 3 h, followed by treatment with AP (50 μg/mL) for 2 h. For measuring mitochondrial membrane potential, we washed the cells with 1× phosphate-buffered saline (PBS) and stained with JC-1 dye (10 μM) for 10 min at 37 ℃ in the dark. After staining, the cells were activated with ATP (5 mM) for 5 min. Images were obtained using a confocal laser-scanning microscope (Carl Zeiss, LSM710).

#### Immunoprecipitation

In a 100pi dish, HEK 293FT cells (2 × 10^6^ cells) were seeded 1 day before the transfection. The cells were transfected with NLRP3-Myc vector (10 μg) using Lipofectamine™ 2000. After 24 h, we treated AP (10 μg/mL, 50 μg/mL, 100 μg/mL) for 2 h, and lysed with lysis buffer (Tris–HCl pH 7.4 30 mM, EDTA 2 mM, NaCl 120 mM, KCl 2 mM, NP-40 0.2%, glycerol 10%, and protease inhibitor cocktail). The lysates were incubated with Myc antibody overnight at 4 ℃, with additional treatment of protein A/G beads for 1 h. The beads were rinsed and eluted. NLRP3-NEK7 binding was analyzed with these samples by immunoblotting.

#### Measurement ATPase activity of NLRP3

Purified mouse recombinant NLRP3 (0.139 mg/mL) was incubated with AP, MCC950, or DMSO in ATPase reaction buffer (100 mM Tris, pH 7.8, 2.8 mM EDTA, 100 mM MgCl_2_, 15 mM KCl, 665 mM NaCl) for 3 h at 25 ℃. Ultra-pure ATP (1 mM) was added for another 40 min at 37 ℃. The ATP hydrolysis by NLRP3 was determined by an ADP-Glo™ Max assay (Promega, Madison, USA) according to the manufacturer’s protocol.

#### ASC oligomerization and ASC speck staining

To investigate ASC oligomerization, pellets of THP-1 cells (1.5 × 10^6^ cells/well in 12 well plate) were resuspended with PBS for washing and incubated with DSS (2.5 mM) for 30 min at 25 ℃. ASC oligomerization was analyzed by immunoblotting.

ASC speck staining was conducted in NLRP3 inflammasome-activated J774A.1 cells (1 × 10^6^ cells/well in 4-well culture slide). The cells were fixed with 4% paraformaldehyde in PBS for 10 min at 25 ℃ and washed thrice with ice-cold PBS with 0.1% Tween-20 (PBS-T). Permeabilization was conducted using PBS containing 0.2% Triton X-100 for 10 min at 25 ℃ and washed three times with ice-cold PBS-T for 5 min. Next, the cells were blocked with 1% bovine serum albumin (BSA) and 22.52 mg/mL glycine in PBS-T and incubated with ASC antibody overnight at 4 ℃. Then, the cells were washed three times with PBS-T for 5 min and incubated with AF488-conjugated anti-mouse IgG antibody in 1% BSA for 1 h at 25 ℃ in the dark. The cells were washed three times with ice-cold PBS-T and cells were mounted in a medium containing DAPI for nuclear staining. A confocal laser-scanning microscope (Carl Zeiss, LSM710) was used for imaging.

### In vivo study

#### Zebrafish husbandry and AP preparations

Zebrafish embryos used in this study are the wild-type TAB5 strain. The embryos were consistently incubated at 28.5 °C. The natural product AP, provided in powder form, is stable at 25 ℃. For experimental use, it was diluted with 500 μL DMSO and 1.5 mL of PBS to achieve a concentration of 10 mg/mL. This solution was then stored at −20 °C.

#### Zebrafish harvest and inflammation induction

Fertilized embryos were harvested and incubated in E3 solution at 28.5 °C. At 1 dpf, embryos underwent a dechorionation procedure. They were then raised in the incubator until further use. The LPS (*Escherichia coli* 055:B5; Sigma) was used to induce inflammation with either method of immersion or injection. Twenty five embryos were placed in a 60 mm petri dish containing 6 mL of solution. After dechorionation, the larvae were treated with E3 solution with 1-phenyl-2-thiourea (PTU) containing 0.125% DMSO and 10 μg/mL LPS and 10 μg/mL AP. The solution in the petri dish was replaced daily. After 48 h, all samples were fixed for subsequent experiments. For the LPS injection method, 3 dpf larvae were anesthetized using tricaine for approximately 2 min. Each of the embryos were injected with 2 nL of either a PBS mixture or 0.5 mg/mL LPS mixture into the yolk. The PBS mixture contained 0.5 μL phenol red, 4.5 μL 1× PBS and the LPS mixture contained 0.5 μL phenol red, 4 μL 1× PBS, and 0.25 μL LPS. Post-injection, all injected larvae were treated with either PTU (DMSO) control or 10 μg/mL AP mixture. 5.5 h post-natural product treatment, samples were fixed samples for the next phase of experiment. For tail cut injury, 3 dpf larvae were anesthetized using tricaine.

#### Sudan black B stain and Whole-mount in situ hybridization (WISH)

All embryos were fixed in 4% paraformaldehyde throughout the night. Sudan black stain method for wounding zebrafish larvae protocols was previously described^65^. For the WISH procedure, the fixed embryos were first washed in PBS and then dehydrated through overnight storage in methanol at −20 °C. Subsequently, the samples were rehydrated using a mixture of methanol and PBS with 0.1% Tween-20, and then permeabilized with acetone at −20 °C. Permeabilized samples were combined with digoxigenin (DIG)-labeled RNA probes in hybridization buffer (50% formamide, 5 × SSC, 500 μg/mL Torula yeast tRNA, 50 μg/mL heparin, 0.1% Tween-20 and 9 mM citric acid (pH 6.5)) were hybridized in a 65 °C water bath. After hybridization, samples were washed using 2× and 0.2× SSC solutions. The washed samples were incubated at 4 °C with alkaline phosphate-conjugated DIG antibodies (1:5000, Roche, Basel, Switzerland). For signal development, samples were mixed with NBT/BCIP substrate (Promega) and alkaline phosphate reaction buffer (100 mM Tris, pH 9.5, 50 mM MgCl_2_, 100 mM NaCl and 0.1% Tween-20) at 25 ℃. Incubate until the signal develops to a certain stage and then stops developing.

### Statistical analysis

Unless stated otherwise, differences between treatment groups were evaluated using one-way analysis of variance (ANOVA) and Tukey’s post hoc test. In the in vitro assay, differences between stimulated and control cells were assessed using the Wilcoxon single-rank test. All statistical analyses were carried out using GraphPad Prism version 8.0.0 for Windows (GraphPad Software, San Diego, California USA, https://www.graphpad.com), and a p-value of < 0.05 was considered significant.

### Ethics approval

All zebrafish experimental protocols and procedures involving the embryos were approved by the Institutional Animal Care and Use Committees at Kyung Hee University Hospital at Gangdong (IACUC: KHNMC AP 2023).

### Supplementary Information

Supplementary Figures.

Supplementary Information.

Supplementary Legends.

## Data Availability

The datasets generated and/or analyzed during the current study are available in the manuscript and figures.
