# Supplementary material for: Aiouea padiformis extract exhibits anti-inflammatory effects by inhibiting the ATPase activity of NLRP3
Source: Sci Rep. 2024 Mar 4;14:5237. doi: 10.1038/s41598-024-55651-z (PMC10909851; doi:10.1038/s41598-024-55651-z)
Supplement: Supplementary file 1 — Supplementary Figures. [file 41598_2024_55651_MOESM1_ESM.pptx]

## Slide 1
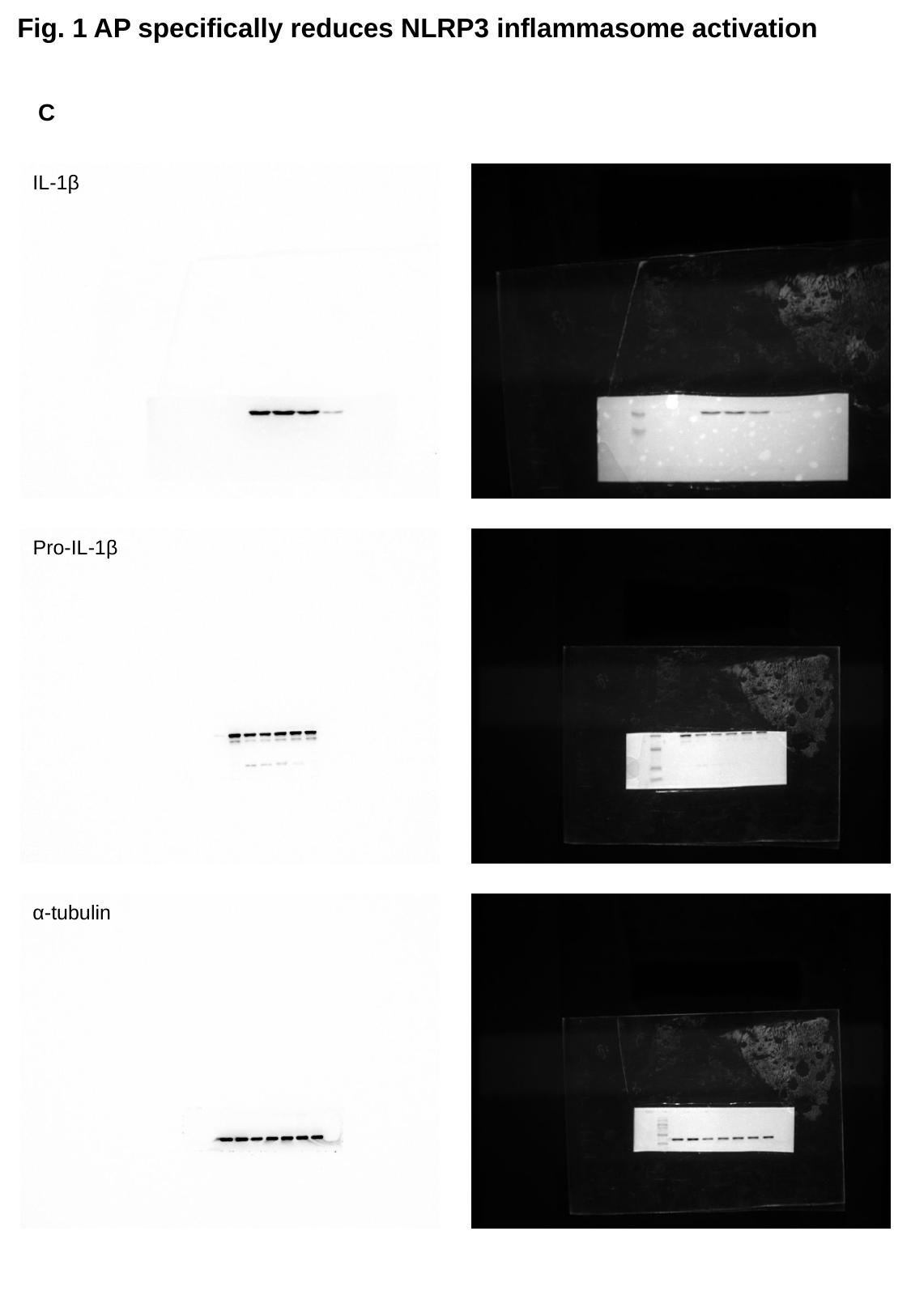

Fig. 1 AP specifically reduces NLRP3 inflammasome activation
C
IL-1β
Pro-IL-1β
α-tubulin

## Slide 2
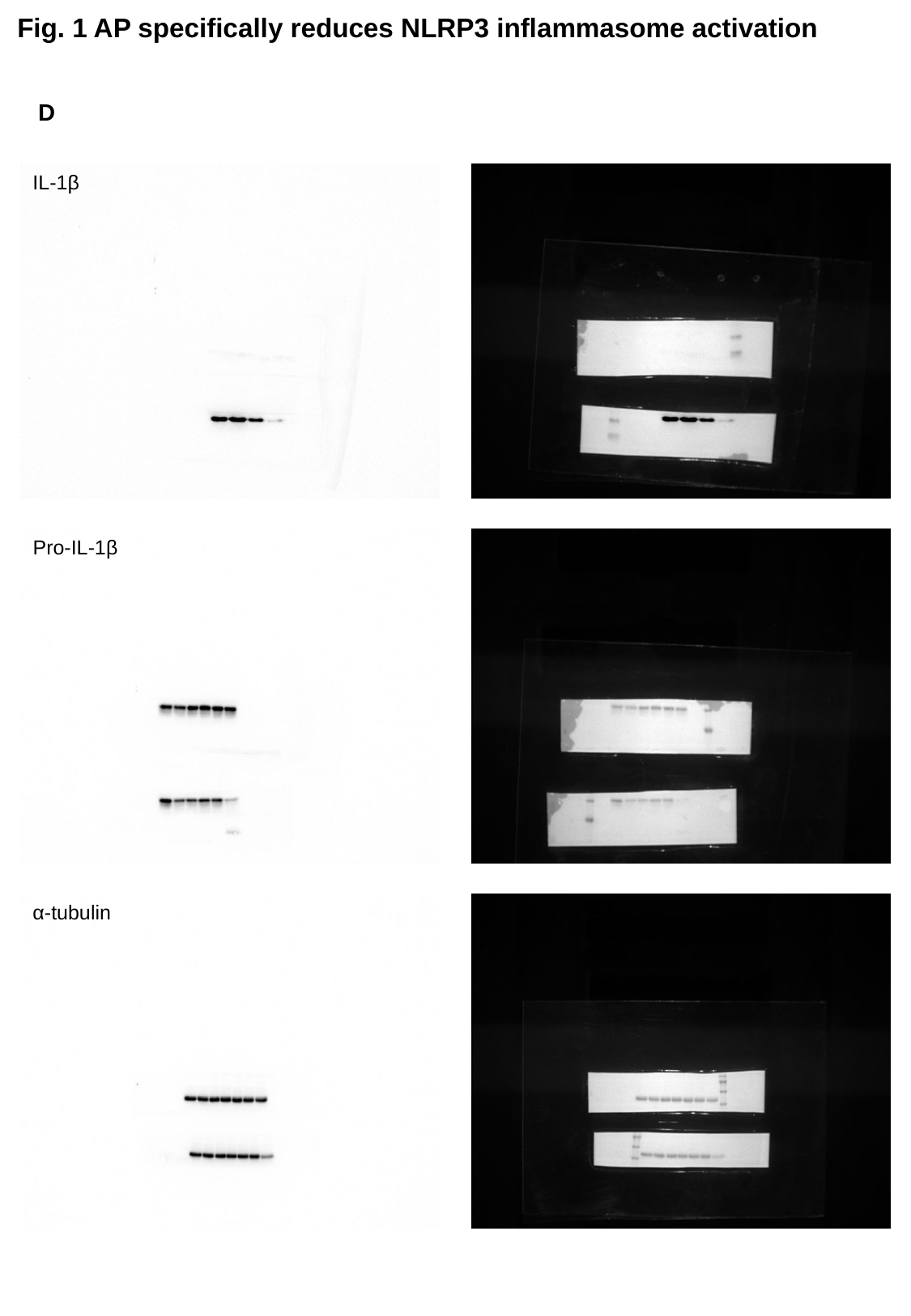

Fig. 1 AP specifically reduces NLRP3 inflammasome activation
D
IL-1β
Pro-IL-1β
α-tubulin

## Slide 3
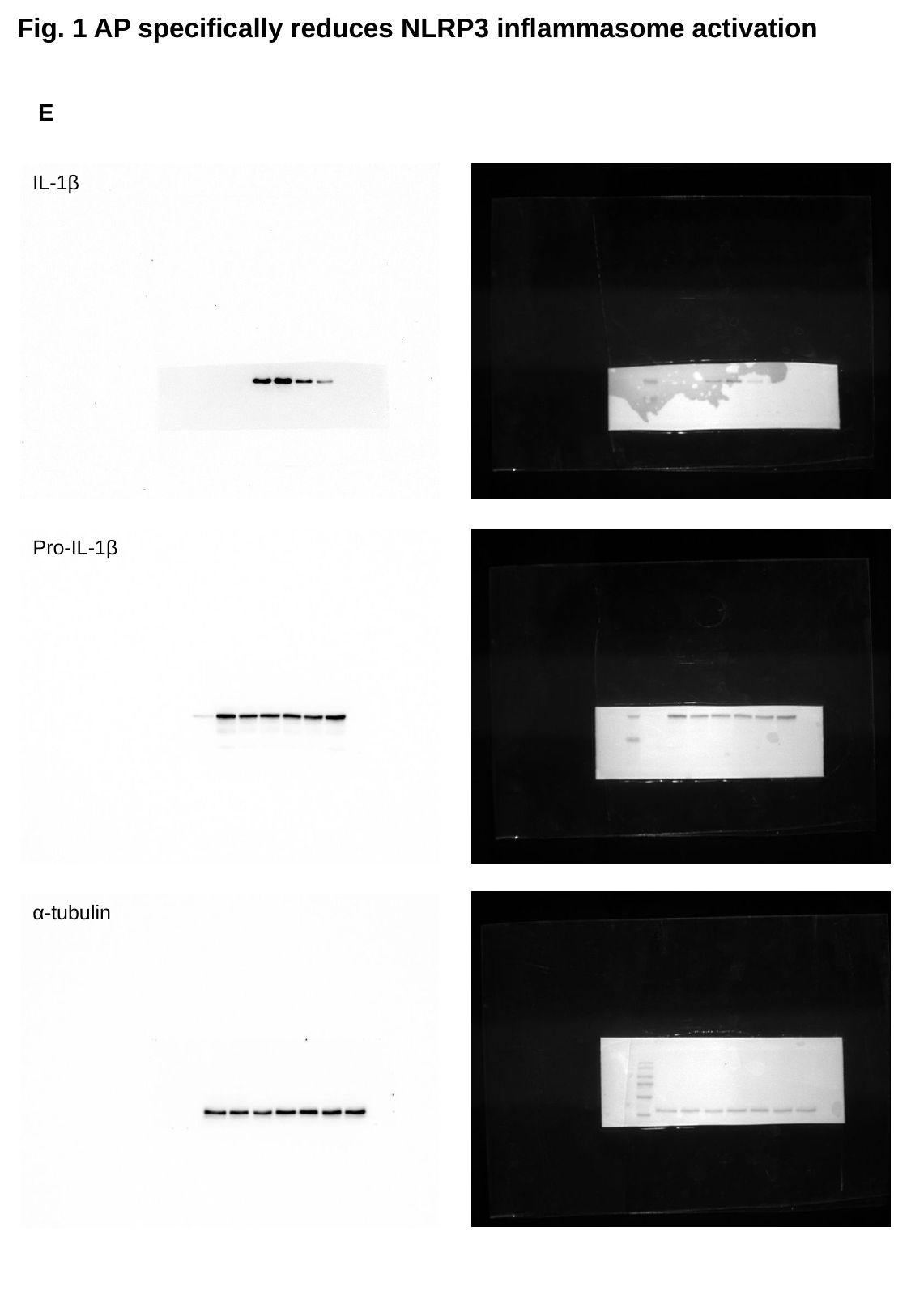

Fig. 1 AP specifically reduces NLRP3 inflammasome activation
E
IL-1β
Pro-IL-1β
α-tubulin

## Slide 4
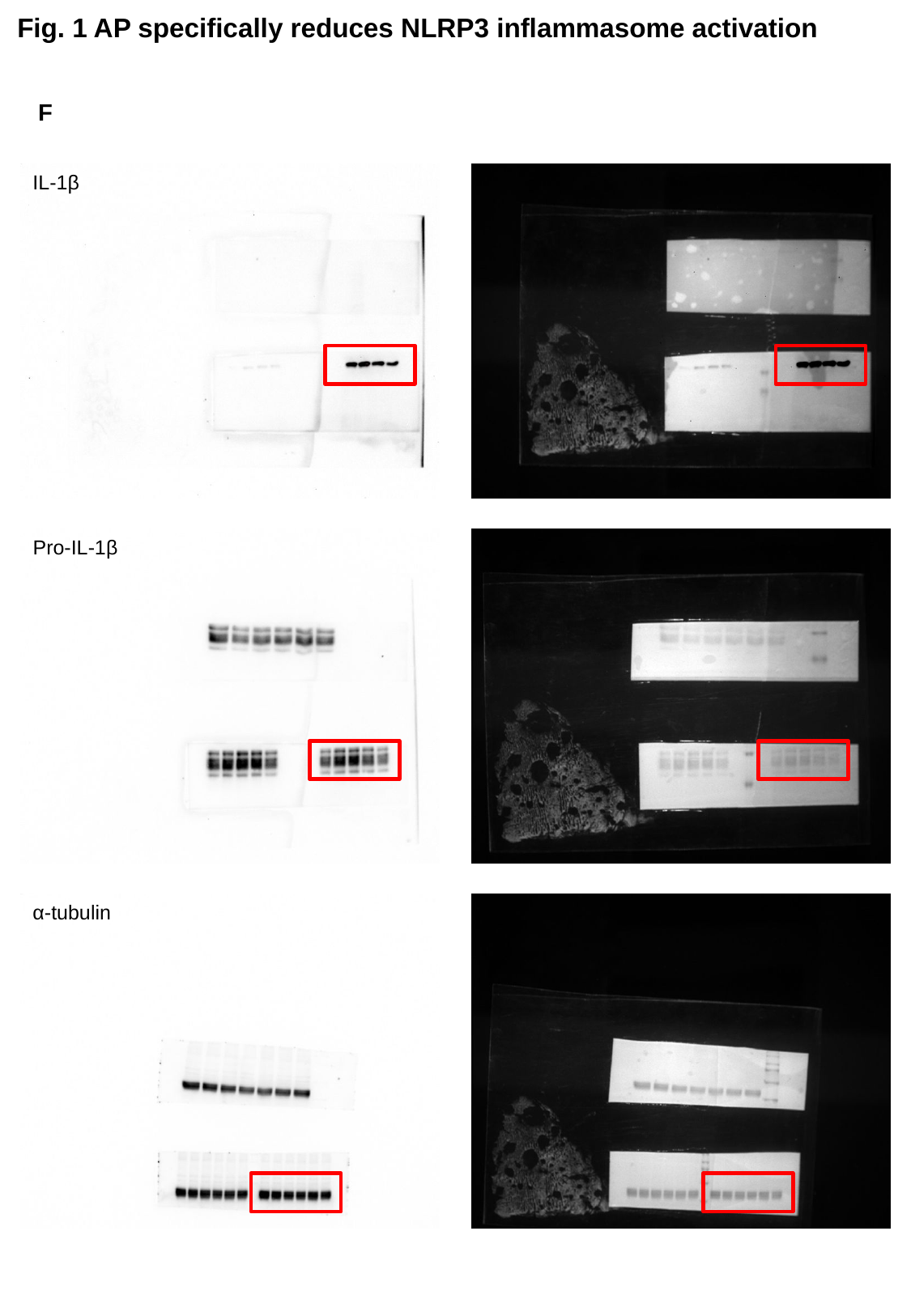

Fig. 1 AP specifically reduces NLRP3 inflammasome activation
F
IL-1β
Pro-IL-1β
α-tubulin

## Slide 5
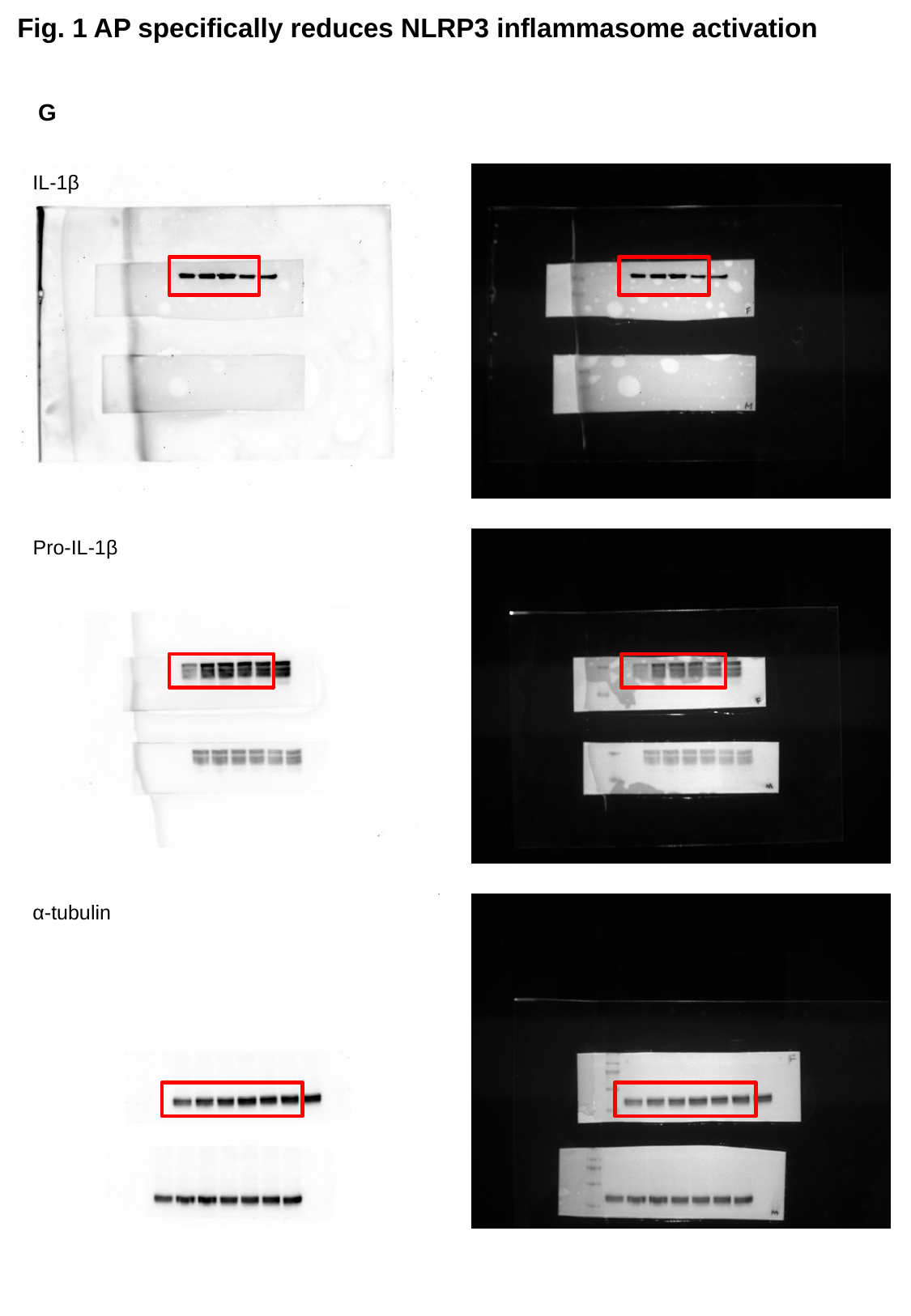

Fig. 1 AP specifically reduces NLRP3 inflammasome activation
G
IL-1β
Pro-IL-1β
α-tubulin

## Slide 6
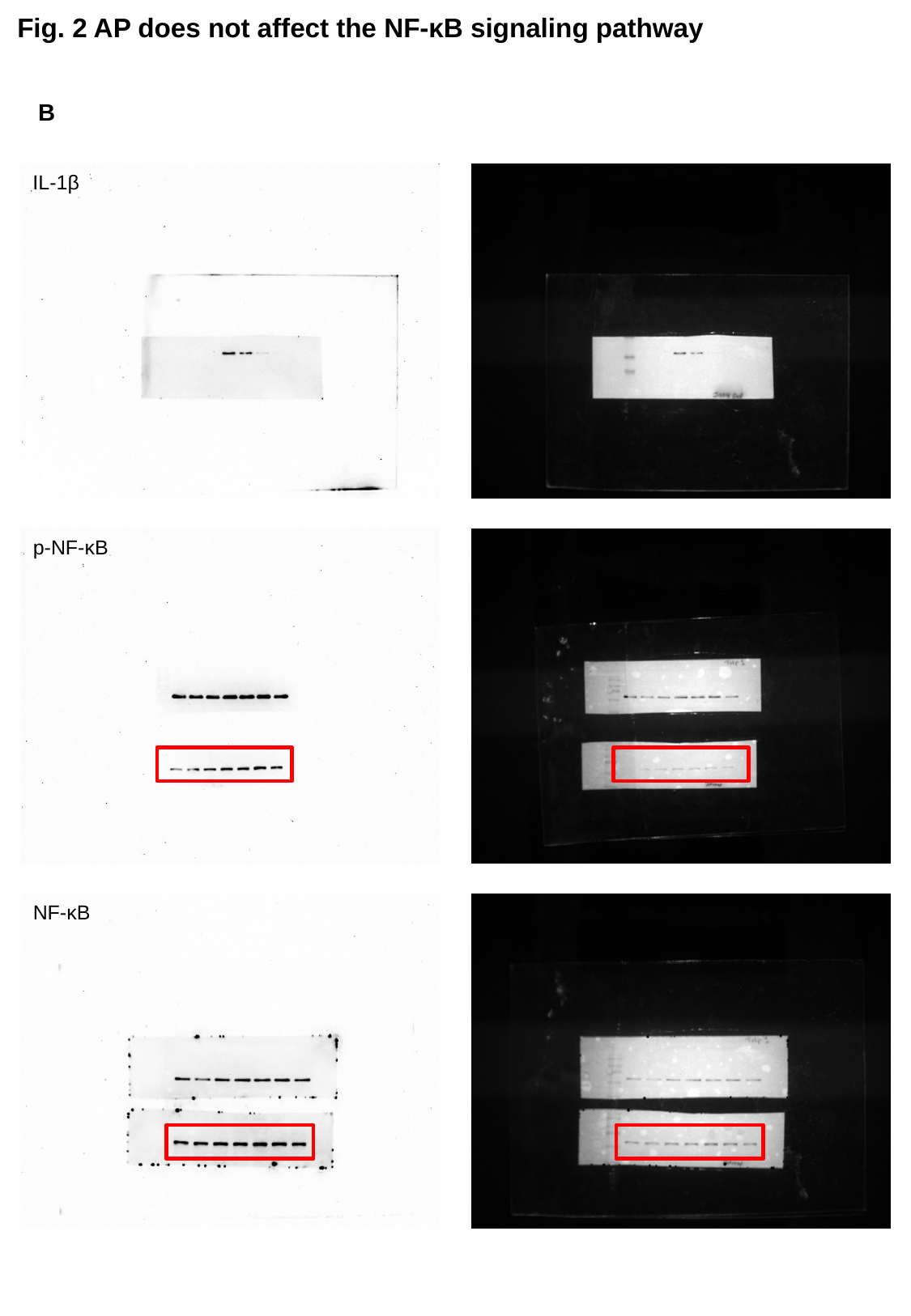

Fig. 2 AP does not affect the NF-κB signaling pathway
B
IL-1β
p-NF-κB
NF-κB

## Slide 7
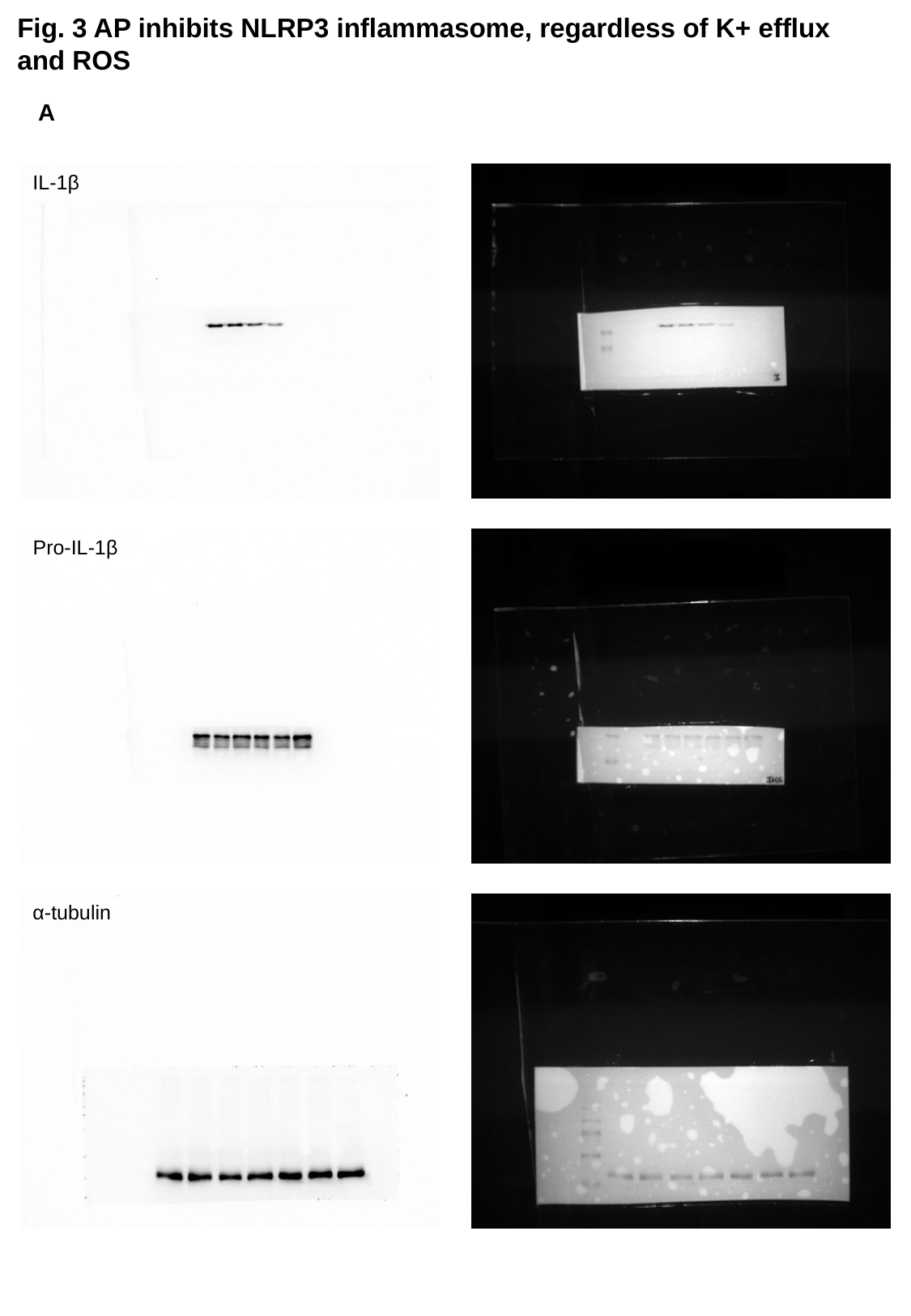

Fig. 3 AP inhibits NLRP3 inflammasome, regardless of K+ efflux and ROS
A
IL-1β
Pro-IL-1β
α-tubulin

## Slide 8
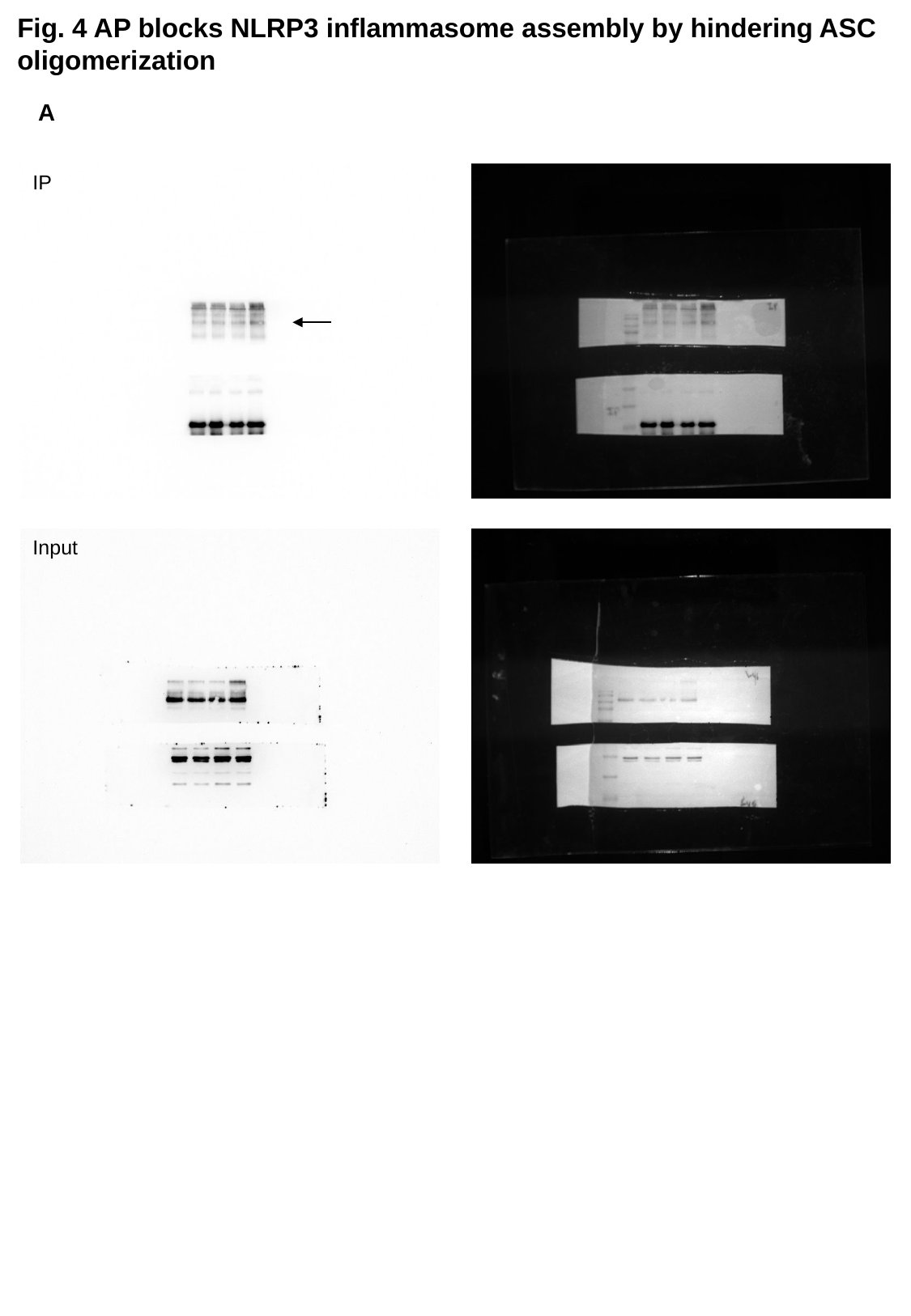

Fig. 4 AP blocks NLRP3 inflammasome assembly by hindering ASC oligomerization
A
IP
Input

## Slide 9
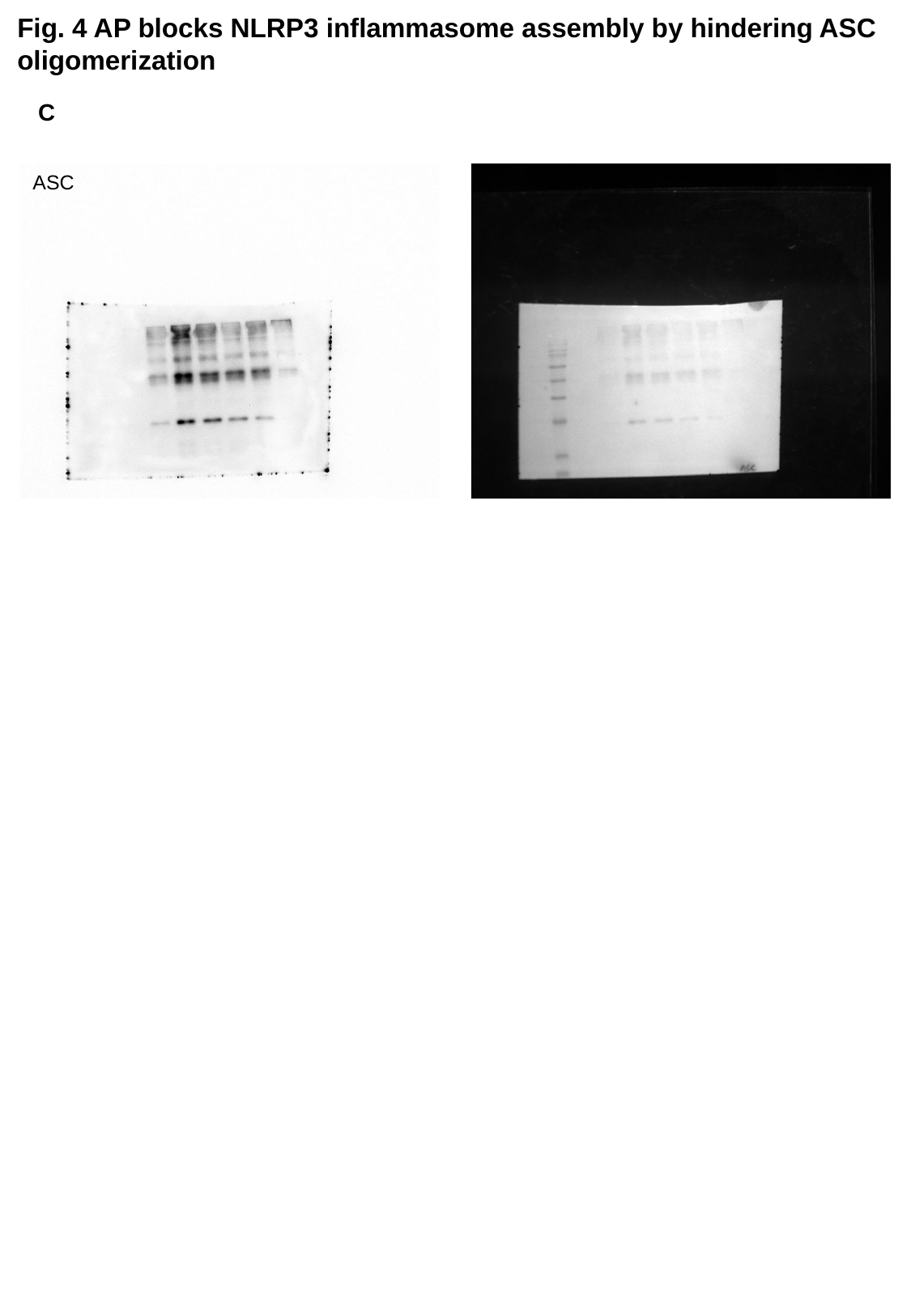

Fig. 4 AP blocks NLRP3 inflammasome assembly by hindering ASC oligomerization
C
ASC
