# Supplementary material for: Aiouea padiformis extract exhibits anti-inflammatory effects by inhibiting the ATPase activity of NLRP3
Source: Sci Rep. 2024 Mar 4;14:5237. doi: 10.1038/s41598-024-55651-z (PMC10909851; doi:10.1038/s41598-024-55651-z)

Supplemental Table 1. Comparison of IL-1β production following plant extracts treatment

No.	Binomial Name	Activator	Activator+4 μg/ml	Activator+40 μg/ml
1	Connarus lentiginosus Brandegee	100	123.8	51.3
2	Alibertia edulis (Rich.) A. Rich.	100	113.3	97.1
3	Combretum fruticosum (Loefl.) Stuntz	100	115.8	92.8
4	Adenopodia patens (Hook. & Arn.) J.R. Dixon ex Brenan	100	126	92.8
5	Trichocentrum ascendens (Lindl.) M.W. Chase & N.H.	100	90.1	11.8
6	Clethra vicentina Standl.	100	176	139
7	Moussonia deppeana (Schltdl. & Cham.) Klotzsch ex Hanst.	100	102.1	23.6
8	Pleopeltis angusta Humb. & Bonpl. ex Willd.	100	147	64.9
9	Gaultheria erecta Vent.	100	164.6	68.7
10	Macleania insignis M.Martens & Galeotti	100	125	144.9
11	Selaginella porphyrospora A.Br.	100	253.1	84.5
12	Govenia liliacea (Lex.) Lindl.	100	202.1	113.3
13	Rubus urticifolius Poir.	100	201.9	77.6
14	Palicourea padifolia (Willd. ex Schult.) C.M.Taylor & Lorence	100	168.6	59.8
15	Hoffmannia nicotianifolia (M.Martens & Galeotti) L.O.Williams	100	109.1	8.9

No.	Binomial Name	Activator	Activator+40 μg/ml	Activator+80 μg/ml
16	Marsdenia veronicae W.D. Stevens	100	39.1	20.4
17	Vachellia farnesiana (L.) Wight & Arn.	100	42.1	53.7
18	Couepia polyandra (Kunth) Rose	100	78.3	101.4
19	Tillandsia pauciflora Sessé & Moc.	100	117	119.4
20	Caesalpinia pulcherrima (L.) Sw.	100	126.8	186.3
21	Trophis mexicana (Liebm.) Bureau	100	177.4	230.2
22	Epidendrum anceps Jacq.	100	209.3	93.2
23	Syngonium podophyllum Schott	100	75.6	36
24	Oreomunnea mexicana (Standl.) J.-F. Leroy subsp. Mexicana	100	149.9	163.7
25	Nectandra salicifolia (Kunth) Nees	100	270.5	274.4
26	Cestrum racemosum Ruiz & Pav.	100	281.4	174.2
27	Croton reflexifolius Kunth	100	191.5	77.5
28	Guadua angustifolia Kunth subsp. Angustifolia	100	252.6	187.2
29	Molinadendron guatemalense (Radlk. ex Harms) P.K.Endress	100	188.3	213.7
30	Columnea rubricaulis Standl.	100	175.9	94.4
31	Schlegelia fuscata A.H.Gentry	100	224.1	162.8
32	Prosthechea brassavolae (Rchb.f.) W.E.Higgins	100	48.5	25.1
33	Clusia stenophylla Standl.	100	136.1	91.9
34	Catopsis paniculata É.Morren	100	87.3	74.9
35	Litsea glaucescens Kunth	100	177.9	183.6
36	Tillandsia leiboldiana Schltdl.	100	100.9	21.6
37	Isocarpha oppositifolia (L.) Cass.	100	102.4	61.1
38	Cantinoa mutabilis (Rich.) Harley & J.F.B.Pastore	100	123.1	97.5
39	Peteravenia schultzii (Schnittsp.) R.M.King & H.Rob.	100	104.8	42.5
40	Dalea tomentosa var. psoraleoides (Moric.) Barneby	100	105	29.3
41	Commelina rufipes Seub.	100	101.8	39.2
42	Aiouea padiformis (Standl. & Steyerm.) R.Rohde	100	76	10.2
43	Staphylea insignis (Tul.) Byng & Christenh.	100	35.6	1.4
44	Rubus miser Liebm.	100	156.6	197.4
45	Damburneya martinicensis (Mez) Trofimov	100	25.4	6.7

Supplemental Figure 1. AP reduces IL-18 secretion mediated by NLRP3 inflammasome

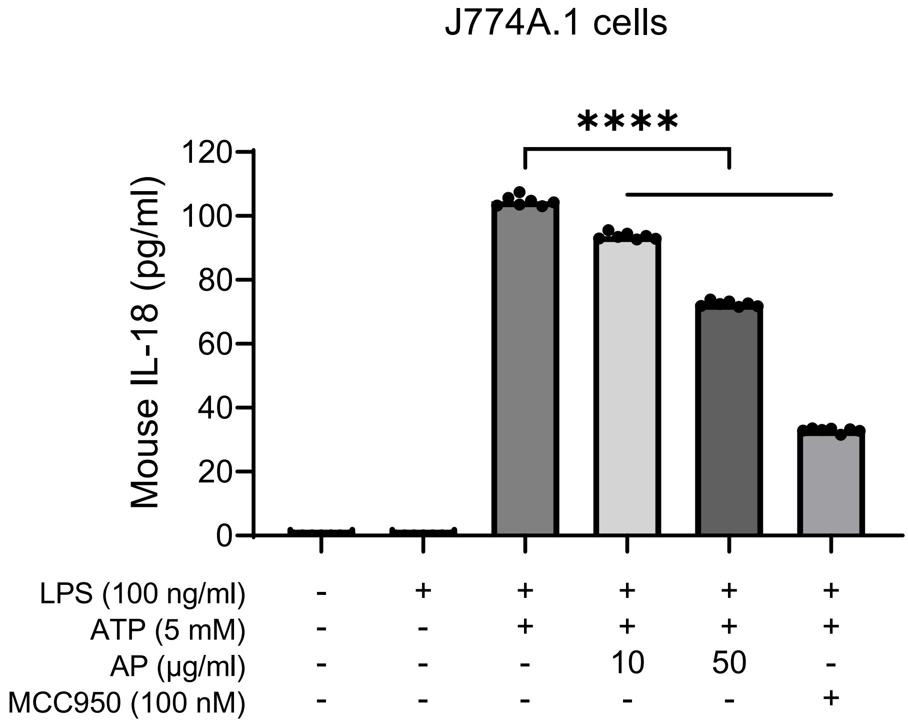

Supplemental Figure 2. Chemical analysis of AP by MPLC and HPLC

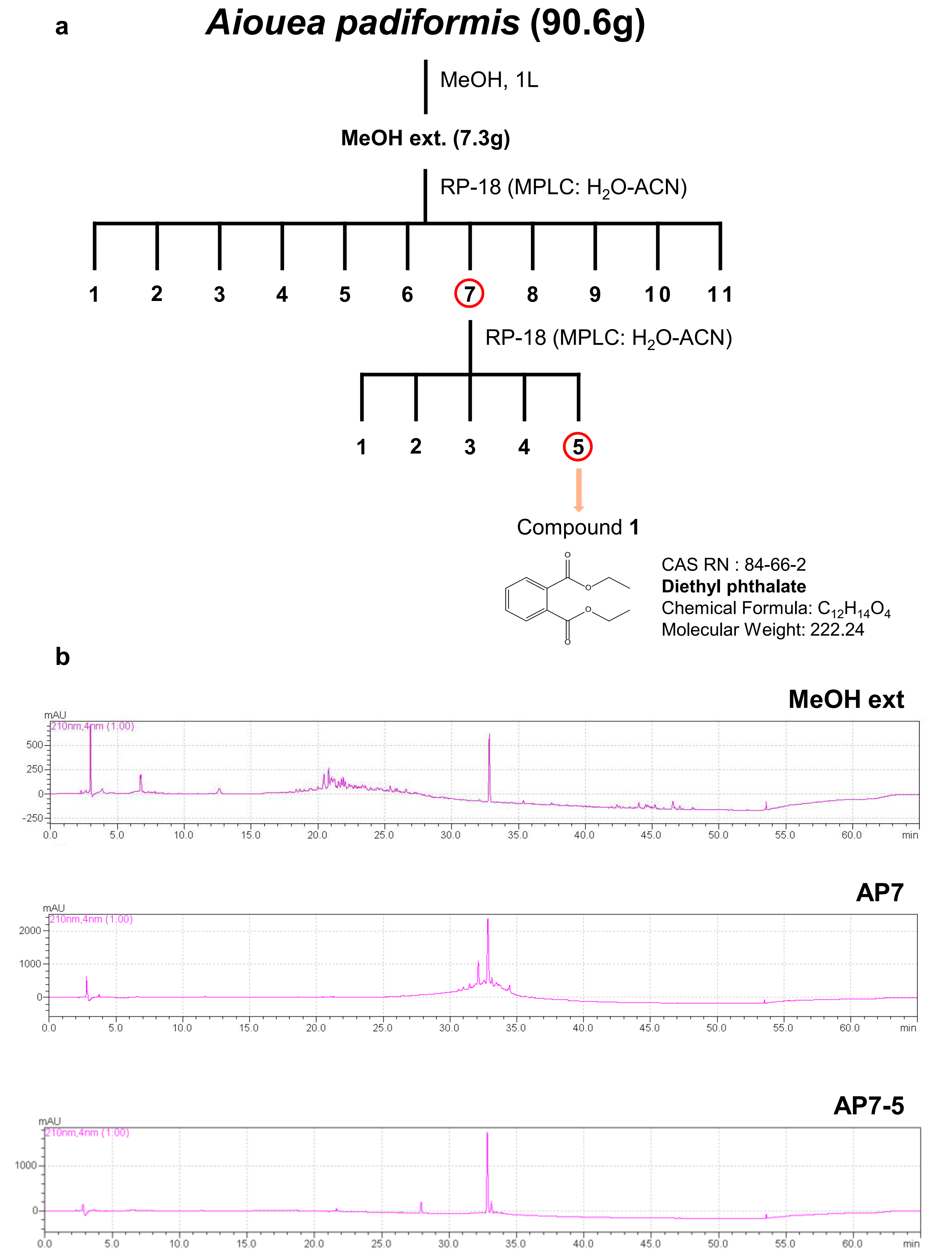

Supplement: Supplementary file 2 — Supplementary Information. [file 41598_2024_55651_MOESM2_ESM.pdf]
