# Supplementary material for: Aiouea padiformis extract exhibits anti-inflammatory effects by inhibiting the ATPase activity of NLRP3
Source: Sci Rep. 2024 Mar 4;14:5237. doi: 10.1038/s41598-024-55651-z (PMC10909851; doi:10.1038/s41598-024-55651-z)
Supplement: Supplementary file 3 — Supplementary Legends. [file 41598_2024_55651_MOESM3_ESM.docx]

**Supplemental table 1. Comparison of IL-1β production following plant extracts treatment**

LPS-primed J774A.1 cells were pretreated with the plant extracts listed in the table, followed by treatment with the NLRP3 activators. The levels of IL-1β were quantified relative to untreated samples (set as 100%) using Western blot with ImageJ (ver. 1.54d).

**Supplemental figure 1. AP reduces IL-18 secretion mediated by NLRP3 inflammasome**

LPS-primed J774A.1 cells were treated with AP for 2 h and activated for 30 min with ATP (5 mM). The level of IL-18 in the supernatants was analyzed by ELISA, measured at a wavelength of 450 nm.

**Supplemental figure 2. Chemical analysis of AP by MPLC and HPLC**

(a) A brief overview of the analysis process involving MPLC and HPLC for AP component analysis and molecular structure of isolated compound elucidated by MS and 1D/2D NMR data analyses. (b) Representative MPLC and HPLC chromatograms of methanolic extract of *Aiouea padiformis*.
